# Therapeutic Potential of Allicin in Multi-System Inflammatory Diseases

**DOI:** 10.3390/cimb48090940

**Published:** 2026-09-15

**Authors:** Jieyou Zhao, Jiawen Zhao, Zhaoyang Li, Nana Cheng, Chenhao Feng, Yunjia Song, Xutao Sun

**Affiliations:** School of Basic Medical Sciences, Heilongjiang University of Chinese Medicine, Harbin 150040, China; zzjtcm@163.com (J.Z.); zhaojiawen0817@163.com (J.Z.);

**Keywords:** allicin, anti-inflammatory mechanisms, NF-κB signaling, NLRP3 inflammasome, Nrf2/HO-1 pathway, multi-system inflammatory diseases

## Abstract

Inflammation is a central pathological process in many chronic diseases and is closely linked to tissue injury and organ dysfunction. Although anti-inflammatory drugs are widely used in clinical practice, their long-term application can be limited by adverse effects and inadequate efficacy, creating a need for safer and more effective therapeutic approaches. Allicin, the principal bioactive organosulfur compound derived from garlic, has attracted increasing research interest because of its anti-inflammatory and antioxidant activities. Preclinical evidence indicates that allicin can attenuate inflammatory responses, inhibit inflammatory cell activation, reduce oxidative stress, and limit tissue damage in various disease models. Protective effects have been reported in models involving the digestive, respiratory, cardiovascular, urinary, and nervous systems. This review summarizes the preclinical evidence for the anti-inflammatory effects of allicin across these organ systems, focusing on the underlying molecular mechanisms, shared signaling pathways, and organ-associated features. The chemical instability, rapid metabolism, and poor oral bioavailability of allicin remain important barriers to clinical translation, while high-quality clinical evidence from studies of purified allicin in humans remains limited. These challenges, together with potential strategies for overcoming them, are also discussed.

## 1. Introduction

Inflammation is a coordinated protective biological response involving immune, vascular, and tissue responses to infection, tissue injury, chemical irritants, sterile insults, and metabolic disturbances, and plays a central role in restoring tissue homeostasis. Well-regulated acute inflammation helps eliminate pathogens and promote tissue repair, whereas excessive, dysregulated, or persistent inflammation can cause tissue damage and contribute to pathological conditions [1,2]. Such chronic low-grade inflammation, marked by sustained leukocyte infiltration and elevated inflammatory cytokines and enzymes [3], is increasingly recognized as a key driver of chronic disease [2,4], including type 2 diabetes [5], atherosclerosis [6], hypertension [7], and irritable bowel syndrome [8].

Current pharmacological options remain limited. NSAIDs suppress prostaglandin biosynthesis via cyclooxygenase inhibition but carry long-term risks of gastrointestinal and cardiovascular injury, while glucocorticoids carry risks of osteoporosis, diabetes, and hypertension, and prolonged treatment may be associated with glucocorticoid resistance or insensitivity in certain diseases or clinical contexts [9,10,11]. These limitations motivate the search for anti-inflammatory agents with multi-target mechanisms, favorable safety, and good accessibility.

Natural products represent one potential source of such agents because of their structural diversity and multi-target activities, although their safety profiles vary substantially depending on the specific compound, dose, duration of exposure, and biological context [12]. Garlic (*Allium sativum* L.) and its principal bioactive organosulfur compound, allicin, exemplify this potential. Allicin, a reactive thiosulfinate, is formed when alliinase converts the precursor alliin upon crushing of the bulb; it is a major contributor to garlic’s characteristic odor and, owing to its lipophilicity, is able to S-thioallylate cysteine residues and react with glutathione inside cells [13].

Allicin has been reported to exert anti-inflammatory, antioxidant, antimicrobial, antineoplastic, and cytoprotective effects through multiple complementary mechanisms. In relation to inflammation, evidence from cellular and animal models indicates that these mechanisms include suppression of the Toll-like receptor 4 (TLR4)/myeloid differentiation primary response protein 88 (MyD88)/nuclear factor-κB (NF-κB) signaling pathway, inhibition of p38 mitogen-activated protein kinase (p38 MAPK) and c-Jun N-terminal kinase (JNK) activity, and activation of the nuclear factor erythroid 2-related factor 2 (Nrf2)/heme oxygenase-1 (HO-1) antioxidant pathway, changes associated with reductions in pro-inflammatory cytokine production [14,15,16]. However, existing reviews have mainly discussed allicin’s anti-inflammatory effects within individual organ systems or alongside its other pharmacological activities, with limited integration of mechanisms across organ systems. This review therefore summarizes the preclinical evidence for allicin’s anti-inflammatory effects across the digestive, respiratory, cardiovascular, urinary, and nervous systems, focusing on shared and tissue-specific mechanisms.

A structured literature search was conducted in PubMed, Web of Science, and CNKI through January 2026. The core search strategy combined terms related to allicin and inflammation, including “allicin,” “garlic,” “*Allium sativum*,” “inflammation,” “inflammatory response,” and “anti-inflammatory,” using Boolean operators (AND/OR). Additional searches incorporated mechanism-, disease-, and organ-specific terms, including “NF-κB,” “NLRP3,” “Nrf2,” “MAPK,” “TLR4,” “MyD88,” and “oxidative stress,” as appropriate for each of the five organ systems examined. Studies were included when they investigated the anti-inflammatory effects or mechanisms of allicin in relevant cellular, animal, or human models. Studies unrelated to allicin or inflammation, duplicate records, and publications without relevant primary or mechanistic evidence were excluded. Reference lists of relevant publications were also screened to identify additional eligible studies. Original research articles constituted the primary evidence base, whereas review articles were consulted mainly for background information and identification of relevant literature.

## 2. Therapeutic Effects of Allicin in Inflammatory Diseases

A critical consideration when interpreting the evidence presented in Section 2, Section 3 and Section 4 is the chemical instability of allicin. Because allicin rapidly degrades and reacts with thiol-containing molecules, the effects reported in vivo or in cell-based studies may reflect the parent compound, its degradation products, or their combined actions. Moreover, the purity, stability, and actual concentration of allicin were not consistently characterized across studies. Therefore, the mechanistic findings summarized below should be interpreted with these limitations in mind.

### 2.1. Inflammatory Diseases of the Digestive System

Inflammatory diseases of the digestive system involve multiple pathological processes, ranging from intestinal mucosal barrier disruption to hepatic inflammatory injury. Available evidence suggests that allicin exerts anti-inflammatory and tissue-protective effects in digestive disease models, with reported changes in canonical inflammatory pathways, gut microbiota composition, intestinal barrier integrity, and gut–liver axis homeostasis.

The intestinal mucosa acts as both a physical barrier and an immune regulator and plays a crucial role in the initiation of digestive inflammation. In an inflammatory bowel disease model, allicin suppressed IL-1β-induced activation of the p38 and JNK pathways in Caco-2 cells and reduced IL-8 and NF-κB p65 expression; in the corresponding in vivo inflammatory bowel disease model, allicin lowered serum TNF-α and IL-1β levels while enhancing IL-4 expression [17]. Allicin also enhanced intestinal barrier function in IPEC-J2 cells, with increased levels of phosphorylated Nrf2 (p-Nrf2) and HO-1 and improved recovery from LPS-induced epithelial damage. Reversal of allicin’s effects on the tight junction proteins ZO-1, occludin, and claudin-1 by the Nrf2 inhibitor ML385 provided additional experimental support for the involvement of Nrf2 signaling in this barrier-protective effect [18]. In an intestinal oxidative injury model, combined treatment with allicin and selenomethionine activated the Nrf2 pathway and alleviated inflammation and oxidative stress, although the lack of an allicin-only group precluded determination of its individual contribution to these effects [19]. Allicin dose-dependently inhibited IκBα degradation and suppressed TNF-α-induced secretion of the pro-inflammatory cytokines and chemokines IL-1β, IL-8, and IP-10 in HT-29 and Caco-2 intestinal epithelial cells, while also reducing their basal secretion under unstimulated conditions, suggesting that the latter effect may reflect reduced basal cytokine production rather than inhibition of an induced inflammatory response. [20]. In an acrylamide-induced intestinal injury model, the protective effects of allicin were linked to alterations in the microbiota–short-chain fatty acid–TLR4/MyD88/NF-κB axis [21].

Beyond the intestine, changes in gut microbiota composition and intestinal barrier function have also been reported alongside the hepatoprotective effects of allicin in models involving the gut–liver axis. In hepatic inflammatory diseases, the reported protective effects of allicin span diverse pathological contexts and involve alterations in multiple signaling pathways. Alcoholic fatty liver disease (AFLD) is a common liver condition caused by chronic alcohol consumption, in which inflammatory responses and lipid metabolic dysregulation interact and contribute to disease progression. In an alcohol-induced liver injury model, allicin treatment was associated with increased GSH levels and catalase (CAT) activity, reduced cytochrome P450 2E1 (CYP2E1) expression, and decreased SREBP-1 expression and hepatic lipid accumulation [22]. Alcohol-induced gut dysbiosis represents an important factor in the progression of AFLD [23]. Allicin altered gut microbiota composition and decreased intestinal permeability, along with reduced LPS translocation into the bloodstream and attenuation of the LPS–CD14–TLR4 signaling pathway. These changes were associated with reduced Kupffer cell activation and lower expression of TNF-α, IL-1β, and IL-6, alongside reduced AFLD-associated inflammation [24]. In metabolic dysfunction-associated steatotic liver disease (MASLD), allicin treatment was associated with changes in the circadian gene Rev-erbα and the recovery of Bmal1 rhythmicity disrupted by a high-fat, high-fructose diet, together with reduced expression of TNF-α, IL-1β, and IL-6. Allicin also altered gut microbiota composition by increasing microbial diversity, reducing the Firmicutes/Bacteroidetes ratio, and increasing the abundance of Akkermansia, Bacteroides, and Lactobacillus. These changes were accompanied by increased production of short-chain fatty acids, including acetate, propionate, and butyrate, together with reductions in hepatic inflammation and macrophage infiltration [25]. Beyond steatotic liver disease, allicin also protects against chemically or pharmacologically induced hepatic inflammation. In acrylamide-treated BRL-3A cells and Kupffer cells, molecular docking predicted that allicin interacts with the CYP2E1 active site; consistent with this, allicin treatment was associated with reduced ROS generation and oxidative stress. Allicin also suppressed ER stress, as reflected by downregulation of GRP78, CHOP, and the IRE1α/TRAF2/p-ASK1/XBP-1s pathway, and these changes were accompanied by attenuated MAPK/NF-κB signaling, NLRP3 inflammasome activation, and release of IL-1β and IL-18 [26]. In a hepatic ischemia/reperfusion injury model, the hepatoprotective effects of allicin were accompanied by changes in the PPARγ/IRAK-M/TLR4 pathway [27]. In cyclophosphamide-induced hepatic injury, allicin reduced iNOS upregulation and the expression of pro-inflammatory cytokines, with concurrent activation of Nrf2-mediated antioxidant response element (ARE) signaling and attenuation of hepatic injury [28].

**Figure 1:**
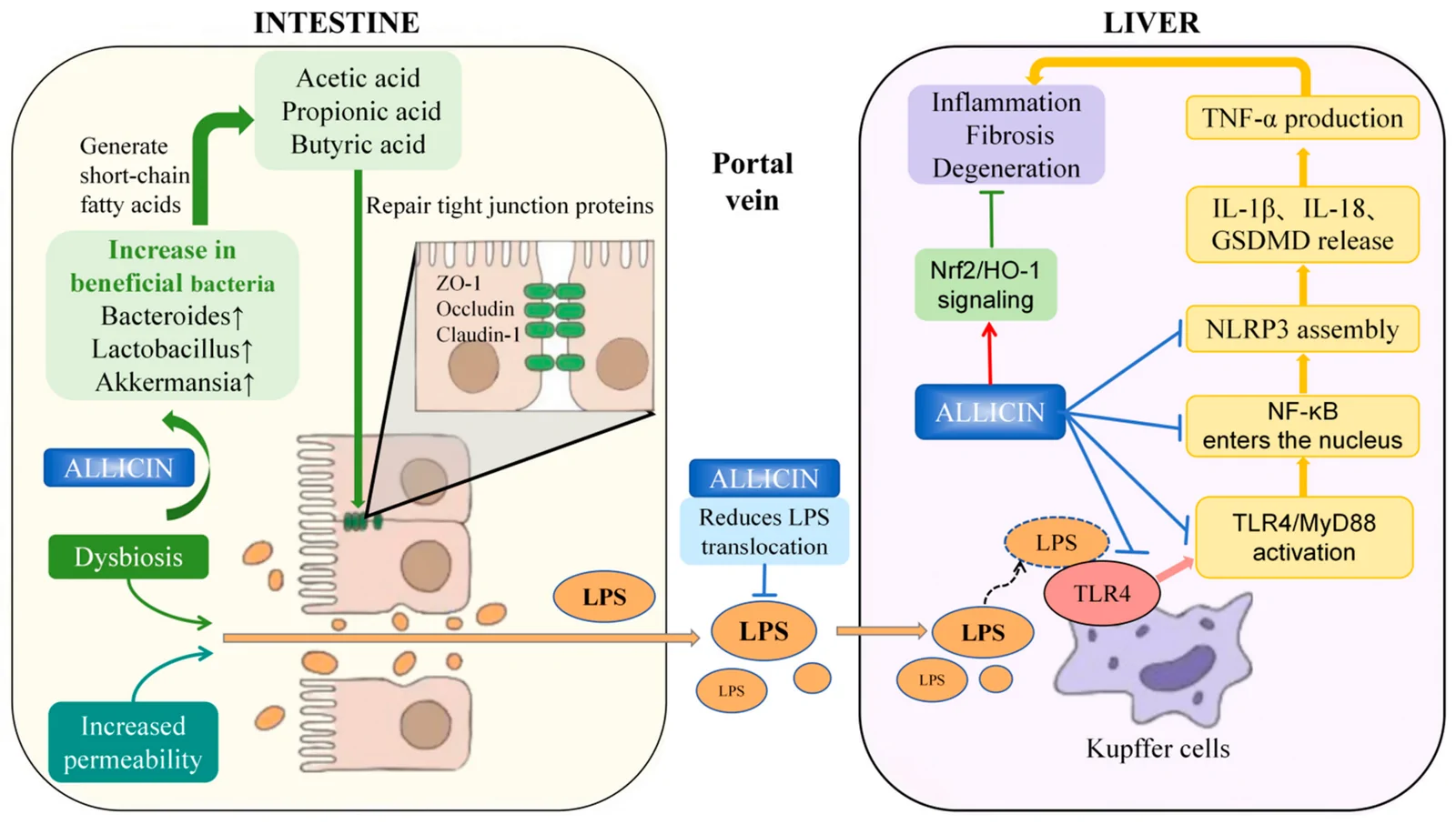
Schematic illustration of allicin’s anti-inflammatory effects in the digestive system via the gut–liver axis.

The anti-inflammatory effects of allicin in the digestive system have been investigated in a variety of injury models, including TNBS-induced colitis, LPS-stimulated intestinal epithelial cells, TNF-α-stimulated Caco-2 and HT-29 cells, acrylamide-induced enteropathy, and alcohol- or diet-induced hepatic steatosis. Although these models involve different injury triggers, inhibition of NF-κB signaling and activation of the Nrf2/HO-1 pathway are recurrent findings. This may suggest a central role for these pathways in the anti-inflammatory effects of allicin, but it may also reflect their frequent use as experimental readouts rather than the existence of a single, unified regulatory cascade. In addition, no individual study has demonstrated the complete microbiota–barrier–liver cascade, and the proposed regulatory framework is largely derived from findings obtained in separate studies. Compared with other organ systems, gut microbiota remodeling, intestinal barrier protection, and gut–liver axis regulation are more frequently reported in digestive diseases and represent prominent features of the current evidence (Figure 1).

Orange arrows indicate LPS translocation across the intestinal barrier into the bloodstream. LPS then travels via the portal vein to the liver and activates TLR4 on Kupffer cells. Yellow arrows indicate the hepatic inflammatory cascade, in which TLR4/MyD88 activation leads to NF-κB nuclear translocation, NLRP3 inflammasome assembly, and release of downstream cytokines. Blue blunt-end lines indicate direct inhibition by allicin of LPS translocation, TLR4/MyD88 activation, NF-κB nuclear translocation, and NLRP3 assembly. Red arrows indicate allicin-mediated activation of Nrf2/HO-1 antioxidant signaling. Green blunt-end lines indicate Nrf2/HO-1-mediated inhibition of inflammation, fibrosis, and degenerative injury. Black dashed arrows indicate the LPS–TLR4 recognition and binding process.

### 2.2. Inflammatory Diseases of the Respiratory System

Respiratory inflammatory diseases contribute substantially to global morbidity and mortality and include a broad spectrum of acute and chronic conditions involving the airways, lung parenchyma, and alveolar–capillary barrier. Acute lung injury (ALI), acute respiratory distress syndrome (ARDS), pulmonary fibrosis, asthma, and infection-associated pneumonia are representative inflammatory respiratory disorders characterized by inflammatory cell infiltration, excessive cytokine release, oxidative stress, and, in some settings, disruption of epithelial or alveolar–capillary barrier integrity. Allicin has been reported to exert anti-inflammatory and protective effects in these conditions through mechanisms that vary across disease settings.

ALI and ARDS involve a complex interplay of pathological processes, including epithelial and endothelial barrier disruption, neutrophil-driven inflammation, and impaired gas exchange. Allicin has demonstrated protective effects across several of these mechanisms. In LPS-induced septic lung injury, allicin reduced bronchoalveolar lavage fluid (BALF) TNF-α, IL-6, and IL-1β levels in a dose-dependent manner, alongside inhibition of the TLR4/MyD88/NF-κB signaling pathway. Allicin also suppressed the apoptosis-related proteins caspase-3 and caspase-9 in lung tissue, alongside mitigation of inflammation and apoptosis [29]. Allicin also downregulated miR-455-3p and restored claudin-4 expression, changes accompanied by improved alveolar epithelial barrier integrity. Allicin also reduced inflammatory mediators and myeloperoxidase (MPO) levels in BALF, with a corresponding reduction in lung tissue injury [30]. These findings suggest that protection of the alveolar barrier may contribute to the anti-ALI effects of allicin.

Pulmonary fibrosis is characterized by progressive interstitial fibrosis. Fibroblast activation and extracellular matrix accumulation involve multiple interacting pathways, among which the transforming growth factor-β1 (TGF-β1)/Smad3 axis plays an important role. Allicin treatment was associated with increased AMPK signaling, reduced TGF-β1/Smad3-related fibrotic responses—including decreased fibroblast-to-myofibroblast transition and extracellular matrix deposition—and attenuated inflammatory cell infiltration and pulmonary fibrosis [31].

The high mortality of invasive pulmonary aspergillosis (IPA) is closely associated with the intense inflammatory response and host immune dysregulation triggered by Aspergillus fumigatus infection [32,33]. Aberrant autophagy activation may contribute to the progression of pulmonary inflammatory responses [34]. Allicin dose-dependently inhibited A. fumigatus spore germination and suppressed intracellular ROS generation, accompanied by reduced ROS/NLRP3 signaling and lower expression of pulmonary inflammatory cytokines. In addition, allicin downregulated microtubule-associated protein 1 light chain 3 beta-II (LC3B-II), reduced autophagosome and autolysosome formation, and was associated with reduced infection-induced autophagy and improved cellular homeostasis [35].

Asthma is a heterogeneous disease with multiple inflammatory phenotypes rather than a single uniform pathology. Much of the existing evidence on allicin relates to the Type 2-high eosinophilic phenotype, in which Th2 cell activation drives overproduction of IL-4, IL-5, and IL-13, leading to eosinophil recruitment, IgE production, and airway hyperresponsiveness. In an asthma model, allicin suppressed STAT3 phosphorylation and downstream PIM1 kinase expression, changes associated with the STAT3/PIM1 axis; these findings were accompanied by reduced aberrant airway smooth muscle cell proliferation and attenuation of TGF-β1-induced fibrotic airway remodeling. Concurrently, allicin reduced eosinophil infiltration in BALF, suppressed Th2 cytokines including IL-4, IL-5, and IL-13, and ameliorated eosinophilic airway inflammation, airway hyperresponsiveness, and structural remodeling [36].

Laryngotracheal stenosis is a pathological narrowing of the larynx, subglottis, and trachea, primarily resulting from intubation injury, radiotherapy, or autoimmune disease, although it can also be idiopathic. Inflammation-mediated fibroblast activation and aberrant collagen deposition are important contributors to disease progression [37,38,39]. Allicin reduced pro-inflammatory cytokines such as TNF-α, IL-1β, and IL-6, while reductions in fibroblast activation and collagen deposition were also observed during abnormal wound healing. Furthermore, allicin coating effectively reduced Staphylococcus aureus adhesion and biofilm formation on tracheal tube surfaces, which was associated with less persistent bacteria-related mucosal inflammation and granulation tissue overgrowth [40].

The protective effects of allicin in respiratory inflammatory diseases extend beyond the suppression of classical inflammatory pathways and may also involve the preservation of alveolar barrier integrity, regulation of airway inflammation, and modulation of tissue remodeling. However, most of the disease models discussed above are supported by only a single primary study, with limited independent replication. Protection of the alveolar barrier, suppression of Th2-mediated inflammation, and antifibrotic activity are therefore among the most frequently reported effects, although further studies are needed to confirm the underlying mechanisms.

### 2.3. Inflammatory Diseases of the Cardiovascular System

Cardiovascular disease (CVD) is the leading cause of death worldwide. Inflammatory responses are present throughout the pathological processes of atherosclerosis, myocardial injury, ischemia/reperfusion injury, and hypertension, constituting a shared molecular pathological basis across these conditions [41,42]. In preclinical models, allicin has been shown to exert protective effects across multiple cardiovascular disease contexts, encompassing improvements in endothelial function, attenuation of oxidative stress, suppression of inflammation, and maintenance of mitochondrial homeostasis. The available evidence, however, derives primarily from in vitro experiments and animal studies; clinical validation remains limited.

Atherosclerosis (AS) is a chronic inflammatory disease of the arterial wall involving endothelial dysfunction, immune cell infiltration, lipid accumulation, and fibrous plaque formation. Endothelial dysfunction is an early and central pathological event in AS development, and oxidative stress together with mitochondrial dysfunction are two key triggers of endothelial abnormality [43,44]. In LPS-injured human umbilical vein endothelial cells (HUVECs), allicin ameliorated oxidative stress injury, reduced inflammatory cytokine release, and attenuated mitochondrial dysfunction, including partial restoration of mitochondrial membrane potential and reversal of LPS-induced alterations in mitochondrial dynamics—characterized by increased expression of the fusion proteins Mfn-1 and Opa-1 and decreased expression of the fission proteins Drp-1 and Fis-1, thereby attenuating endothelial inflammation [45]. In a high-fat diet-induced coronary atherosclerosis mouse model, allicin activated the eNOS/Nrf2/HO-1 pathway in endothelial progenitor cells (EPCs), alongside increased NO production and EPC survival and migration, and improvement in vascular endothelial repair [46]. In addition, allicin promoted cholesterol reverse transport via ABCA1 activation, alongside improvement in endothelial dysfunction, potentially reducing one important driver of vascular inflammation at an early stage of atherosclerotic lesion development [47].

During myocardial ischemia/reperfusion, excessive ROS generation and pro-inflammatory immune cell infiltration cause secondary myocardial injury [48]. Mechanistically, allicin activated PI3K signaling, which was associated with reduced phosphorylation of GRK2, CaMKII, PLC-γ, and IP3R and attenuated intracellular Ca^2+^ overload; pharmacological activation of PLC with m-3M3FBS reversed this effect, supporting a role for the PI3K/GRK2/PLC-γ/IP3R axis in allicin’s protection against reperfusion-induced myocardial injury [49]. In a complementary in vitro hypoxia/reoxygenation model using primary cardiomyocytes, allicin reduced apoptosis, inflammatory cytokine release, and ROS generation, and attenuated the loss of mitochondrial membrane potential, alongside upregulation of PGC1-α and eNOS, and downregulation of HIF-1α, endothelin-1 and TGF-β [50].

Doxorubicin (DOX) is an effective antitumor drug, but its clinical application is significantly restricted because of its cardiotoxicity [51]. DOX-induced cardiac injury is closely associated with oxidative stress, inflammatory responses, and cardiomyocyte apoptosis. Allicin attenuated oxidative stress in cardiac tissue, as evidenced by reduced MDA and 8-OHdG levels, elevated glutathione (GSH), and enhanced antioxidant enzyme activity, and was also associated with reduced NO levels. The increased NO following DOX exposure was attributed by the authors to possible iNOS upregulation based on previous studies; however, the study did not directly assess eNOS or iNOS expression or activity, and the measured NO therefore could not be attributed to a specific source. Accordingly, the reduction in NO with allicin treatment should be interpreted with caution rather than as confirmed evidence of reduced iNOS-derived NO. Allicin treatment was also associated with lower IL-1β, TNF-α, and myocardial injury marker levels, including aspartate aminotransferase (AST), lactate dehydrogenase (LDH), creatine kinase (CK), and creatine kinase-MB (CK-MB), as well as preservation of mitochondrial membrane potential, reduced caspase-3-mediated cardiomyocyte apoptosis, and improved cardiac tissue structure and function [52].

Sepsis-associated myocardial dysfunction is a common and life-threatening complication of sepsis, closely associated with excessive free radical generation and cytokine storms [53,54]. In LPS-injured cardiomyocytes, allicin was associated with activation of the Nrf2–HO-1 pathway and suppression of NLRP3 inflammasome activity. Allicin promoted Nrf2 nuclear translocation and upregulated HO-1 expression, enhancing myocardial antioxidant capacity and reducing MDA levels, while increasing superoxide dismutase (SOD) activity and scavenging excess free radicals. Concurrently, allicin markedly suppressed NLRP3 inflammasome activation, blocked caspase-1 cleavage, and reduced the maturation and release of IL-1β and other inflammatory mediators; these changes were accompanied by attenuation of cardiomyocyte injury and cardiac dysfunction in this LPS model [55].

Pulmonary arterial hypertension (PAH) is primarily a pulmonary vascular disease characterized by progressive loss and obstructive remodeling of the pulmonary vascular bed, leading to stepwise increases in pulmonary arterial pressure and vascular resistance, and ultimately to cardiac dysfunction and right heart failure. It is discussed in the cardiovascular section because PAH can impose a substantial hemodynamic burden on the right heart and lead to progressive right heart dysfunction [56]. Allicin mitigated perivascular inflammatory cell infiltration and lowered TNF-α, IL-6, and IL-1β levels. It also attenuated pulmonary vascular fibrosis and remodeling by downregulating TGF-β and alpha-smooth muscle actin (α-SMA), suggesting that it may modulate both vascular inflammation and fibrosis [57].

Diabetic cardiomyopathy is a major contributor to heart failure and cardiovascular mortality in diabetic patients, characterized by myocardial fibrosis and inflammation and manifesting primarily as cardiac hypertrophy, arrhythmia, and heart failure [58]. In a study using allicin in combination with artemisinin, treatment was associated with reduced expression of NF-κB p65 and phosphorylated NF-κB p65 in the myocardial tissue of rats with diabetic cardiomyopathy. These changes were accompanied by reduced inflammatory mediator production and decreased NADPH oxidase-dependent ROS generation. The combined treatment was also associated with reduced cardiomyocyte injury and apoptosis and attenuation of myocardial fibrosis [59].

The cardiovascular anti-inflammatory effects of allicin involve several mechanisms that are relatively well characterized among the organ systems covered in this review. Liver X receptor alpha (LXRα)/ABCA1-mediated reverse cholesterol transport has been reported predominantly in cardiovascular models; whether this reflects genuine tissue specificity or simply the current research focus of the literature remains to be clarified. Allicin has been reported to activate LXRα and promote ABCA1-mediated cholesterol efflux, which may reduce foam cell formation and thereby attenuate the lipid-driven inflammatory signaling associated with atherosclerosis. This upstream lipid–inflammation cross-regulatory pathway has not yet been reported in the other organ systems covered in this review, although the absence of such reports may reflect the scope of available studies rather than a definitive absence of the pathway in those tissues. Allicin’s mitochondrial protective effects in the cardiovascular system are also relatively well-characterized. Allicin has been reported to preserve mitochondrial membrane potential and reduce mitochondria-derived ROS and apoptotic signaling in cardiovascular disease models [45,50,52]. In one study, allicin also increased the expression of the fusion proteins Mfn-1 and Opa-1 while reducing the fission proteins Drp-1 and Fis-1 [45], suggesting a potential role in regulating mitochondrial dynamics. However, whether this effect is shared across other cardiovascular disease models remains unclear. Given the central role of mitochondrial oxidative phosphorylation in cardiomyocytes, mitochondrial protection may be particularly relevant in cardiovascular diseases. Similar mechanisms in other organ systems may simply be less well studied.

### 2.4. Inflammatory Diseases of the Urinary System

Acute kidney injury (AKI) and chronic kidney disease (CKD) represent a substantial portion of the global disease burden, with oxidative stress and inflammatory responses acting as major contributors to progressive renal functional decline [60]. Evidence from experimental animal and in vitro models indicates that allicin exerts notable protective effects across multiple kidney disease models, spanning pathological contexts including acute injury, metabolic nephropathy, drug-induced nephrotoxicity, and infectious disease; clinical evidence in this area remains limited.

Sepsis-associated AKI is among the most common organ dysfunctions in the course of sepsis and carries high morbidity and mortality [61]. Allicin improved survival in a cecal ligation and puncture (CLP)-induced sepsis mouse model and ameliorated renal function by reducing serum creatinine, blood urea nitrogen (BUN), urinary albumin (UALB), and the injury markers kidney injury molecule-1 (KIM-1) and neutrophil gelatinase-associated lipocalin (NGAL). A major mechanism involves activation of the Nrf2/HO-1 pathway, promoting Nrf2 nuclear translocation and upregulating HO-1 expression, which in turn reduces IL-6, IL-1β, TNF-α, and monocyte chemoattractant protein-1 (MCP-1) levels in the renal cortex, decreases ROS and MDA accumulation, and enhances antioxidant enzyme activity. ML385 reversal experiments provided additional evidence for the involvement of Nrf2 signaling in this renoprotective effect [62].

Renal ischemia/reperfusion injury (IRI) is a common pathophysiological event during kidney transplantation, although its severity varies considerably and is influenced by organ preservation techniques, and represents a major risk factor for early delayed graft function and long-term chronic graft failure [63]. Allicin enhanced renal antioxidant capacity by increasing SOD activity and reducing MDA levels, alongside improvement in IRI-induced renal dysfunction. Allicin also inhibited tubular epithelial cell apoptosis, as reflected by decreased Bax and cleaved caspase-3 expression, increased Bcl-2 expression, and reduced cytochrome-c release, consistent with attenuation of mitochondrial dysfunction-associated apoptotic signaling. In addition, allicin upregulated the cytokines IL-4 and IL-10, which are broadly associated with anti-inflammatory and immunoregulatory functions, while downregulating IL-6 and TNF-α, alongside attenuation of local renal inflammation [64].

Drug-associated nephrotoxicity is a common form of renal injury encountered in clinical practice. Allicin ameliorated cisplatin-induced renal injury in rats, suppressing abnormal elevations in serum creatinine, urea, and uric acid and correcting electrolyte disturbances. Allicin protected against nephrotoxicity by enhancing renal antioxidant defenses and reducing inflammation. It increased renal GSH levels and the activities of glutathione peroxidase, SOD, and CAT, while decreasing MDA and NO levels. Allicin also reduced TNF-α levels in both serum and renal tissue. The decrease in NO was proposed to result from reduced TNF-α-mediated iNOS induction, although iNOS expression was not directly examined [65]. In gentamicin-induced nephrotoxicity, allicin intervention reduced renal MDA, myeloperoxidase (MPO), nitric oxide metabolites (NOx, comprising nitrite and nitrate) and TNF-α levels, and restored GSH levels and SOD activity, along with decreased serum creatinine and increased creatinine clearance, thereby attenuating renal injury through both antioxidant and anti-inflammatory effects [66].

Allicin has also been reported to exert renoprotective effects in the context of metabolic syndrome (MS)-associated renal injury and in diabetic nephropathy (DN), the latter representing a major form of chronic kidney disease. Allicin reversed MS-induced overexpression of IL-1β, IL-6, and TNF-α in the renal cortex. At the pathway level, allicin suppressed NF-κB activation and increased IκBα expression by approximately 2.5-fold, consistent with prevention of NF-κB nuclear translocation and subsequent pro-inflammatory gene transcription [15]. Concurrently, allicin enhanced antioxidant defenses in MS-induced renal injury by increasing Nrf2 expression, promoting its nuclear translocation, and restoring SOD and GPx activities. The authors suggested that the anti-inflammatory effects of allicin may also involve crosstalk between the Nrf2 and NF-κB pathways. However, this interaction was inferred from previous studies and was not directly examined in their study [15]. DN is one of the most common chronic inflammatory renal diseases encountered in clinical practice, representing a progressive complication of diabetes that is particularly refractory to reversal in its later stages and is associated with high incidence throughout the course of the disease. The pathogenesis of DN is closely linked to inflammatory cell infiltration and aggravated oxidative stress. Allicin reduced the levels of inflammatory mediators including C-reactive protein (CRP), IL-1β, IL-6, IL-18, and TNF-α, and attenuated renal fibrosis, as reflected by downregulation of TGF-β1 expression. These anti-inflammatory effects were accompanied by reduced renal lipid peroxidation (4-hydroxynonenal, 4-HNE) and protein oxidation (DNPH-reactive carbonyls), together with decreased NF-κB expression and increased expression of its inhibitor IκB, consistent with attenuation of the reciprocal amplification between oxidative stress and NF-κB-mediated inflammatory signaling implicated in DN progression [67].

Urinary tract infection (UTI) is a high-incidence infectious disease of the urinary system, with Escherichia coli being the predominant causative pathogen in uncomplicated, community-acquired cases; complicated and healthcare-associated UTIs involve a broader range of pathogens [68]. One mechanism through which allicin has been reported to intervene in UTI involves regulation of the mucosa-associated lymphoid tissue lymphoma translocation protein 1 (MALT1)/protein kinase B (AKT)/NF-κB signaling axis, in which MALT1 acts as an upstream adaptor protein. Allicin suppressed aberrant activation of MALT1 and the AKT/NF-κB pathway, reducing IL-6 and IL-1β expression; phosphoinositide 3-kinase (PI3K) inhibitor pretreatment and MALT1-small interfering RNA (siRNA) transfection experiments further supported the notion that the MALT1/AKT/NF-κB axis is an important pathway through which allicin exerts its anti-inflammatory effects in this context [69].

The protective effects of allicin in urinary diseases are mainly associated with reduced inflammation and oxidative stress and have been reported in models of acute kidney injury, chronic kidney disease, drug-induced nephrotoxicity, and urinary tract infection. The mechanisms involved, however, differ among these conditions. Nrf2/HO-1 and NF-κB signaling have been implicated in several forms of renal injury, whereas the MALT1/AKT/NF-κB axis has been described mainly in UTI, indicating that the effects of allicin may vary with the underlying disease context. Most of the available evidence is derived from individual animal or cell-based studies, and direct comparisons between different renal diseases remain limited. Thus, preservation of renal function, reduction in oxidative and inflammatory injury, and attenuation of renal fibrosis are the main effects reported to date, although their relative contributions and relevance to human renal disease remain unclear.

### 2.5. Inflammatory Diseases of the Nervous System

Neuroinflammation plays a complex, context-dependent role in the pathophysiology of numerous neurological conditions: while an acute, transient inflammatory response can support tissue repair and pathogen clearance, sustained or excessive neuroinflammation is a key driver of secondary injury and neurodegeneration in conditions such as traumatic brain injury (TBI), cerebrovascular disease, neurodegenerative diseases, and exogenous neurotoxicity. The interaction among microglia-mediated neuroinflammation, oxidative stress, and neuronal apoptosis is a common pathological feature across these conditions. By targeting multiple inflammatory and oxidative stress signaling pathways, attenuating mitochondrial dysfunction, and inhibiting abnormal immune cell activation, allicin has been reported to exert neuroprotective effects in various neurological disease models.

#### 2.5.1. Acute Neurological Injury

TBI encompasses primary and secondary injury mechanisms that collectively lead to neurological deficits and inflammatory responses. Microglia-driven neuroinflammation is a key secondary injury mechanism after TBI and may further aggravate neurological damage [70,71]. Allicin increased protein kinase C delta (PKC-δ) expression, facilitating plastin 3 (PLS3) phosphorylation at threonine 21 (Thr21), which promoted cardiolipin transport and externalization from the inner to the outer mitochondrial membrane during TBI. Although increased PKC-δ activity has been associated with neuroinflammation and neuronal injury in other disease contexts, its role appears to be context-dependent. In this TBI model, the PKC-δ/PLS3 axis was instead associated with cardiolipin-dependent mitophagy, facilitating the clearance of damaged mitochondria and reducing mitochondria-associated inflammatory signaling. Consistent with this interpretation, pharmacological inhibition or knockdown of PKC-δ attenuated allicin-induced mitophagy and diminished its protective effects. Allicin treatment was also associated with reduced levels of the pro-inflammatory cytokines TNF-α, IL-1β, and IL-6, together with downregulation of the inflammasome/signaling components NLRP3 and TLR4 in brain tissue. Separately, allicin reduced ROS levels, indicating attenuated oxidative stress [72]. Thus, the apparent discrepancy between these findings and the generally deleterious effects attributed to PKC-δ may reflect differences in cellular context and downstream signaling, although the precise determinants of this context-dependent response remain to be established.

Beyond TBI, the anti-inflammatory and antioxidant effects of allicin have also been investigated in spinal cord injury (SCI), another form of traumatic central nervous system injury. In a spinal cord injury model, allicin activated the Nrf2 signaling pathway, promoting its nuclear translocation in neurons and astrocytes, which enhanced antioxidant capacity and mitigated oxidative stress. Allicin treatment was associated with reduced pro-inflammatory cytokine levels at the injury site and improved neurological outcomes [73].

Intracerebral hemorrhage (ICH), a type of hemorrhagic cerebrovascular disease, involves bleeding into the brain parenchyma, leading to inflammation and oxidative stress, which exacerbate brain injury and neurological dysfunction [74,75]. Allicin was associated with reduced immune cell activation and recruitment in the brain, as evidenced by decreased microglia/macrophage accumulation around the hemorrhagic lesion and reduced neutrophil infiltration into the hematoma core. Allicin also downregulated the mRNA expression of pro-inflammatory cytokines and chemokines in brain tissue, and these changes were associated with attenuated post-ICH neuroinflammation and reduced neurological deficits [76].

In subarachnoid hemorrhage (SAH), allicin reduced the lipid peroxidation product MDA, restored endogenous antioxidant enzyme SOD activity, and recovered GSH levels. It inhibited caspase-3 activation and reduced neuronal apoptosis, as assessed by Nissl and TUNEL staining. These changes coincided with reduced BBB permeability, as demonstrated by the Evans blue extravasation assay, attenuated brain edema, and improved neurological outcomes [77].

Ischemic stroke results from cerebral arterial occlusion leading to brain infarction and is commonly accompanied by injury to neurons, astrocytes, and oligodendrocytes [78]. Allicin was associated with reduced serum TNF-α levels and inhibited MPO activity, accompanied by attenuated inflammatory responses following cerebral ischemic injury [79]. Because serum TNF-α is a peripheral inflammatory marker, these findings may not directly reflect neuroinflammation within the brain. In addition, allicin upregulated the deubiquitinase OTUD1, which was associated with increased Nrf2 deubiquitination and reduced Nrf2 degradation. This was accompanied by enhanced Nrf2 nuclear translocation, reduced neuronal mitochondrial oxidative stress, and alleviation of cerebral ischemia/reperfusion injury [80].

#### 2.5.2. Neurodegenerative Diseases

Neurodegenerative diseases are commonly accompanied by chronic neuroinflammation, in which aberrant microglial activation, oxidative stress, and neuronal apoptosis interact with one another and contribute to disease progression. Alzheimer’s disease (AD) is marked by Aβ plaque accumulation, tau hyperphosphorylation, oxidative stress, and neuroinflammation [81,82]. Chronic activation of microglia leads to the release of pro-inflammatory cytokines such as IL-1β and TNF-α, along with free radicals, L-glutamate, and prostaglandin E2, exacerbating neuronal damage and cell death [83]. Allicin mitigated oxidative stress, as indicated by enhanced SOD activity and decreased MDA levels, and was associated with reduced phosphorylation of JNK and c-Jun. It also regulated the expression of the mitochondria-related proteins Bax and Bcl-2, paralleled by attenuated neuronal apoptosis and injury [84].

In a metal-induced neurotoxicity model with AD-like pathological features, combined aluminum and copper exposure disrupted metal ion homeostasis and was associated with hippocampal neuroinflammation and cognitive decline. Allicin attenuated hippocampal inflammation associated with metal toxicity in a dose-dependent manner, as reflected by reduced IL-1β, IL-6, and TNF-α levels, restored glutathione levels, and reduced lipid peroxidation. These changes were accompanied by decreased Aβ deposition and improved cognitive function in rats with AD-like pathological features [85].

In a model of natural aging-induced cognitive decline, hippocampal Nrf2 expression and its downstream targets NQO1 and γ-GCS were significantly reduced compared with young controls. These changes were accompanied by decreased GPx activity, GSH levels, and total antioxidant capacity, as well as increased lipid peroxidation (TBARS), protein oxidation, and ROS accumulation in aged animals. Allicin treatment increased Nrf2 expression and its downstream antioxidant targets, restored antioxidant capacity, and attenuated oxidative damage, with improvements in spatial learning and memory performance in the Morris water maze, reflected by shorter escape latency and increased time spent in the target quadrant [86].

#### 2.5.3. Secondary Neurological Injury and Neurotoxicity

Beyond primary neurological diseases, allicin has also been reported to exert protective effects in models of secondary neurological injury and neurotoxicity.

Hepatic encephalopathy is a severe neurological complication of acute or chronic liver failure, characterized by neurological and cognitive dysfunction, and can represent a life-threatening condition [87]. Allicin dose-dependently restored liver function and reduced elevated inflammatory mediators, including TNF-α and IL-1β, as well as oxidative stress biomarkers in both the liver and brain. These changes were associated with attenuated neuroinflammation and oxidative injury in acute liver failure [88].

Acrylamide neurotoxicity is closely associated with oxidative stress and neuroinflammation. Excessive ROS generation disrupts redox homeostasis in brain tissue and is associated with inflammatory cytokine release, neuronal injury, and multi-organ damage [89]. Allicin enhanced brain antioxidant capacity, as reflected by increased GSH levels and reduced glutathione disulfide (GSSG) levels, accompanied by attenuated oxidative stress-related inflammation. Reduced inflammatory cytokine release and inflammatory signaling pathway activation were associated with reduced inflammation-related neurotoxicity. Allicin also increased acetylcholine, serotonin, and dopamine levels in brain tissue following acrylamide exposure and reduced acetylcholinesterase activity. These changes may reflect altered neurotransmitter metabolism and were accompanied by reduced neuroinflammation [90].

Although depression is primarily classified as a neuropsychiatric disorder rather than a classical inflammatory neurological disease, neuroinflammation and oxidative stress are increasingly recognized as contributors to its pathophysiology. Therefore, depression-related models provide complementary evidence for the potential neuroprotective effects of allicin. In animal models of depressive-like behavior, allicin was associated with reduced microglial activation, inflammatory cytokine expression, and ROS accumulation in the hippocampus, together with decreased MDA and protein carbonyl levels and increased SOD activity and Nrf2 signaling. Allicin also suppressed NLRP3 inflammasome activation and reduced the expression of related components, changes that were associated with improved depressive-like behavior [91].

Current studies have linked the neuroprotective effects of allicin to modulation of inflammation, oxidative stress, mitochondrial homeostasis, and neuronal survival. However, this evidence comes almost exclusively from experimental animal and cell-based models, with clinical evidence in neurological populations currently lacking. Moreover, most of the conditions discussed above—including TBI, SCI, ICH, SAH, ischemic stroke, hepatic encephalopathy, acrylamide neurotoxicity, and depressive-like behavior—are currently supported by a single primary study, with little independent replication. The mechanisms reported in AD models are also heterogeneous, involving JNK/c-Jun, PERK/Nrf2, p38 MAPK, and antioxidant responses, but these pathways have been investigated separately in different experimental models. Whether they represent complementary mechanisms or model-specific effects remains unclear. PKC-δ/PLS3-mediated mitochondrial quality control, the OTUD1/Nrf2 axis, and PERK/Nrf2 signaling have likewise been reported in individual neurological models, but their broader relevance to allicin action in the nervous system has yet to be determined. Overall, current evidence supports a role for allicin in modulating neuroinflammation and oxidative stress while preserving mitochondrial and neuronal function. Further studies with independent validation are needed to determine whether these mechanisms are broadly applicable or restricted to specific disease contexts.

The studies summarized in Table 1 are representative examples selected to illustrate the range of models and mechanisms discussed above and do not constitute an exhaustive compilation of all published evidence on this topic.

## 3. Core Mechanisms of the Anti-Inflammatory Effects of Allicin

The regulation of inflammatory signaling networks involves multiple interconnected pathways, and aberrant activation of these pathways contributes to chronic inflammatory diseases. Current evidence suggests that the anti-inflammatory effects of allicin are not attributable to a single molecular target. Instead, allicin’s anti-inflammatory effects have been linked to modulation of multiple signaling pathways involved in the inflammatory response. Allicin has been reported to affect the initiation of inflammation, signal amplification, and tissue repair across different disease models. This section discusses the molecular mechanisms underlying the anti-inflammatory effects of allicin from four perspectives, the TLR4/MyD88/NF-κB signaling axis, the NLRP3 inflammasome, the Nrf2/HO-1 antioxidant–anti-inflammatory axis, and the MAPK pathway, and further examines the crosstalk among these pathways.

### 3.1. Suppression of the TLR4/MyD88/NF-κB Signaling Pathway

The NF-κB signaling pathway plays a central role in regulating the transcription of inflammatory genes [92,93]. Specific pathogen-associated molecular patterns (PAMPs) and damage-associated molecular patterns (DAMPs) are recognized by distinct pattern recognition receptors (PRRs), including TLR4, which recognizes a subset of these danger signals and can activate the MyD88-dependent signaling pathway. This leads to activation of the IκB kinase (IKK) complex, which phosphorylates IκBα and promotes its subsequent degradation, thereby allowing the NF-κB p65 subunit to translocate into the nucleus and promote the transcription of pro-inflammatory mediators such as TNF-α, IL-1β, IL-6, and IL-8 [93].

Evidence predominantly from in vitro and animal studies indicates that allicin can inhibit this pathway at various levels. Allicin has been reported to suppress TLR4/MyD88 signaling, prevent IκBα degradation, and reduce NF-κB p65 phosphorylation and nuclear translocation. These changes were accompanied by decreased expression of downstream pro-inflammatory cytokines. In various experimental models, inhibition of the TLR4/MyD88/NF-κB axis has been frequently associated with the anti-inflammatory effects of allicin, based largely on pathway-level associations rather than definitive target validation [20,21,27,29].

### 3.2. Regulation of the NLRP3 Inflammasome

The NLRP3 inflammasome is a key multiprotein complex in innate immunity that links the sensing of danger signals to the maturation and release of pro-inflammatory cytokines. Its core components include NLRP3, apoptosis-associated speck-like protein containing a CARD (ASC), and pro-caspase-1. Upon activation by PAMPs or DAMPs, the NLRP3–ASC–pro-caspase-1 complex promotes caspase-1 activation, leading to the maturation of IL-1β and IL-18 and the induction of pyroptosis through gasdermin D (GSDMD) cleavage [94]. Aberrant activation of the NLRP3 inflammasome has been associated with a range of inflammatory diseases, including cardiovascular, metabolic, gastrointestinal, and neurodegenerative diseases [94,95].

Current evidence suggests that allicin may modulate NLRP3 inflammasome activation through multiple upstream mechanisms. Allicin has been reported to inhibit NF-κB signaling, reduce ROS accumulation, attenuate endoplasmic reticulum stress, and activate the Nrf2/HO-1 pathway. These changes have been associated with reduced NLRP3 inflammasome priming and activation, as well as decreased caspase-1 activation and IL-1β and IL-18 production [26,55,96]. However, most studies provide associative rather than direct evidence that allicin inhibits NLRP3 at the molecular level. In addition, growing evidence suggests that gut microbiota remodeling may contribute to NLRP3 inflammasome inhibition, suggesting that the microbiota–inflammation axis may be involved in the anti-inflammatory effects of allicin [96]. In summary, inhibition of the NLRP3 inflammasome represents an important downstream mechanism by which allicin links oxidative stress attenuation to the suppression of inflammatory cytokine production.

### 3.3. Activation of the Nrf2/HO-1 Antioxidant–Anti-Inflammatory Axis

Nrf2 is an important regulator of cellular antioxidant defense mechanisms. Under basal conditions, Nrf2 is retained in the cytoplasm through its interaction with Kelch-like ECH-associated protein 1 (Keap1). In response to oxidative stress or electrophilic stimuli, Nrf2 dissociates from Keap1, translocates to the nucleus, and binds to the antioxidant response element (ARE), thereby inducing the expression of cytoprotective genes, including HO-1, NAD(P)H quinone oxidoreductase 1 (NQO1), and glutamate–cysteine ligase catalytic subunit (GCLC) [62]. The Nrf2/HO-1 axis may therefore link oxidative stress and inflammatory signaling through its antioxidant and cytoprotective effects.

Allicin has been shown to activate the Nrf2/HO-1 pathway and promote Nrf2 nuclear translocation, although direct in vivo evidence for Keap1 modification remains limited [15,18,62,97]. Activation of Nrf2 signaling enhances antioxidant defense capacity and may reduce ROS accumulation, which may in turn limit the expression of inflammatory mediators. In LPS-stimulated cardiomyocytes, siRNA-mediated Nrf2 knockdown abolished allicin’s suppressive effect on NLRP3 protein expression, providing direct evidence that Nrf2 activation is required for allicin-induced NLRP3 inhibition in this model; however, as Nrf2 knockdown was only assessed in combination with allicin treatment, this finding demonstrates that Nrf2 is necessary for allicin’s effect on NLRP3 rather than establishing an allicin-independent regulatory relationship between the two pathways [55,62]. Nrf2 signaling has also been reported to negatively regulate NF-κB activity and NLRP3 inflammasome activation in other pathological contexts, such as cerebral ischemia–reperfusion injury, suggesting potential crosstalk among these pathways that may extend beyond allicin’s specific effects [15,18,19,45,55,62]. Taken together, these findings suggest that Nrf2/HO-1 activation may contribute to the antioxidant and anti-inflammatory effects of allicin.

### 3.4. Inhibition of the MAPK Pathway (JNK/p38)

Mitogen-activated protein kinases (MAPKs) are important mediators of inflammatory signaling and cellular stress responses [98]. Within the MAPK family, JNK and p38 MAPK are closely associated with inflammatory responses, although extracellular signal-regulated kinase (ERK) signaling also contributes to inflammation in many cell types. These MAPK pathways contribute to pathological processes, including inflammatory gene expression, immune cell activation, and tissue injury. Activation of these pathways promotes the production of pro-inflammatory mediators such as TNF-α and IL-6, and may also contribute to NLRP3 inflammasome activation [99,100].

In specific experimental models, allicin has been reported to inhibit MAPK signaling, as evidenced by reduced phosphorylation of JNK and p38 MAPK. Allicin reduced the production of pro-inflammatory mediators and may indirectly suppress NF-κB activation and NLRP3 inflammasome signaling. These findings suggest that MAPK inhibition may contribute to the anti-inflammatory and protective effects of allicin in the experimental models studied [17,26,99].

### 3.5. An Integrated View of Multi-Pathway Interactions

The anti-inflammatory effects of allicin appear to involve the coordinated regulation of multiple interconnected signaling pathways rather than intervention at a single target. These pathways are functionally interconnected and can influence the intensity and duration of inflammatory responses. However, the relative contribution and functional interactions of these signaling networks may vary substantially depending on cell type and disease context. NF-κB activation promotes the transcription of inflammasome-related genes, including NLRP3 and pro-IL-1β, thereby contributing to the priming step required for subsequent inflammasome activation [101,102]. JNK1 further promotes inflammasome activation at the post-translational level through phosphorylation of NLRP3 at Ser194 [103]. These mechanisms may act together to facilitate NLRP3 inflammasome activation.

Meanwhile, p38 MAPK enhances the stability of pro-inflammatory cytokine mRNAs containing AU-rich elements, including TNF-α and IL-6, through its downstream target MAPK-activated protein kinase 2 (MK2), thereby amplifying inflammatory signaling at the post-transcriptional level [104]. Accumulation of TNF-α can further activate NF-κB, forming a positive feedback loop that amplifies inflammation [102]. In contrast, activation of the Nrf2/HO-1 pathway can limit ROS accumulation and may thereby reduce ROS-dependent activation of p38 MAPK and the NLRP3 inflammasome [105]. However, this relationship has not been directly demonstrated in most of the cited studies [95,106]. These findings suggest that allicin may modulate several interconnected inflammatory pathways, acting at multiple signaling nodes rather than a single target.

The broad regulatory effects of allicin are likely related to its chemical properties. As a reactive organosulfur compound, allicin can covalently modify cysteine-containing proteins through S-thioallylation, a modification that adds a 72 Da mass shift to reactive cysteine thiols [107,108]. A proteomic survey using LC-MS/MS identified 332 S-thioallylated proteins in human Jurkat T cells, including cytoskeletal, chaperone, and metabolic proteins [107], supporting the potential multi-target nature of this modification. Because cysteine-reactive electrophiles can modify redox-sensitive regulatory proteins such as Keap1, allicin has been proposed to activate Nrf2 through Keap1 modification; however, this interaction has not been directly demonstrated for allicin and remains speculative [62,97]. Similarly, although redox-sensitive cysteines in NF-κB pathway components such as IKKβ and p65 can be modified by other thiol-reactive electrophiles, direct evidence for their modification by allicin is currently lacking. Thus, S-thioallylation provides a plausible basis for the multi-target effects of allicin, but the direct molecular targets involved in its anti-inflammatory activity and their functional contribution remain incompletely understood. Further studies using chemical proteomics and target-validation approaches are needed to establish these relationships.

**Figure 2:**
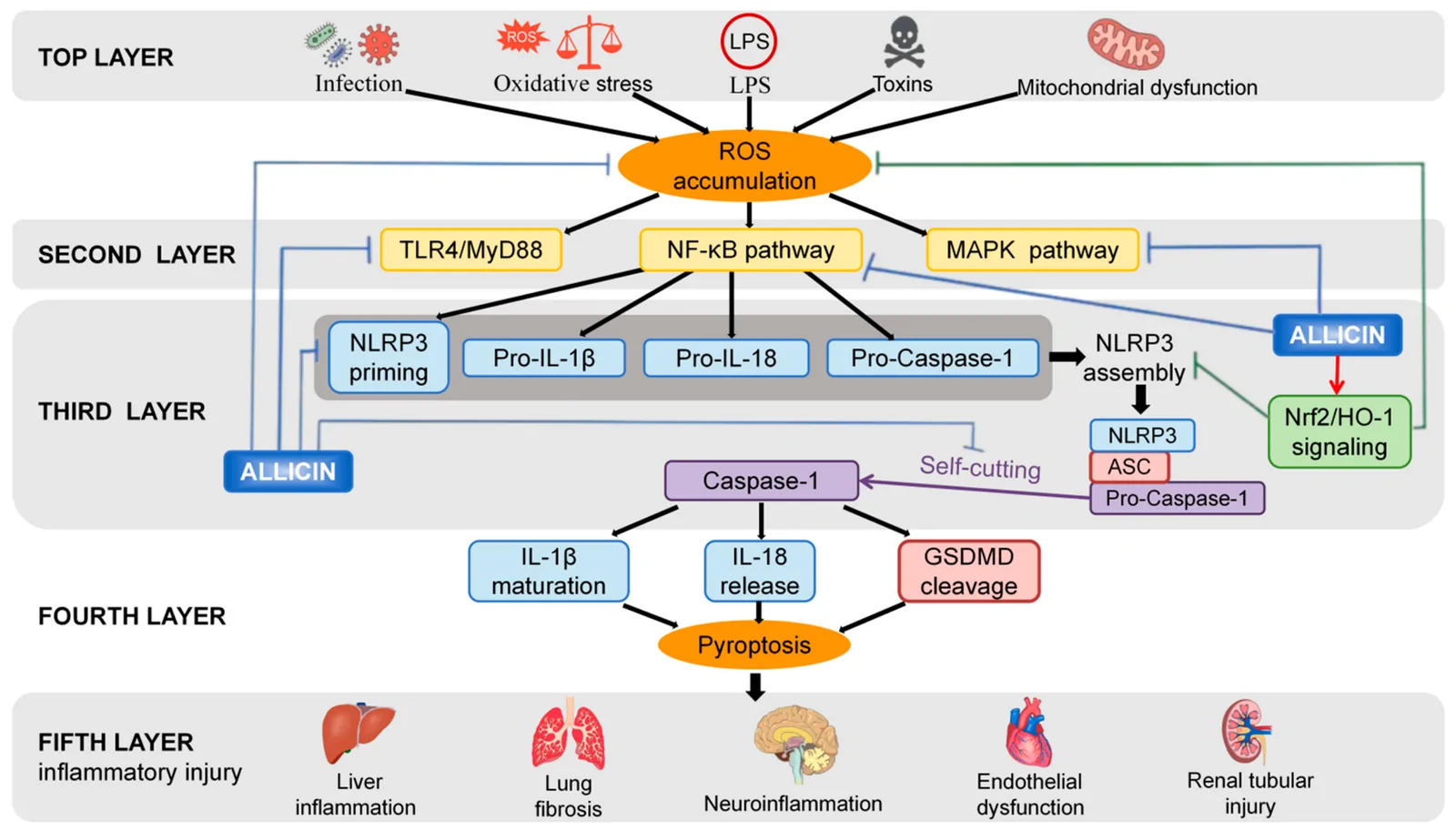
Hierarchical mechanism of allicin’s suppression of the inflammatory cascade.

Such multi-target activity may be relevant to chronic inflammatory conditions, although its therapeutic significance remains to be established. This multi-pathway activity may partly explain the protective effects of allicin reported in the digestive, respiratory, cardiovascular, urinary, and nervous systems, although the available evidence is derived predominantly from preclinical studies (Figure 2).

Upstream stimuli—including infection, oxidative stress, lipopolysaccharide (LPS), toxins, and mitochondrial dysfunction—converge on reactive oxygen species (ROS) accumulation, which activates three parallel signaling pathways: TLR4/MyD88, NF-κB, and MAPK. These pathways provide priming signals that upregulate NLRP3, pro-IL-1β, pro-IL-18, and pro-caspase-1, enabling assembly of the NLRP3–ASC–pro-caspase-1 inflammasome complex. Within this complex, pro-caspase-1 undergoes proximity-induced autocatalytic cleavage into active caspase-1, which mediates IL-1β maturation, IL-18 release, and gasdermin D (GSDMD) cleavage, collectively driving pyroptosis and downstream organ-specific inflammatory injury (e.g., liver inflammation, lung fibrosis, neuroinflammation, endothelial dysfunction, and renal tubular injury). Black arrows indicate activation; blue blunt-end lines indicate direct inhibition by allicin, targeting ROS accumulation, TLR4/MyD88, NF-κB, MAPK, and NLRP3 priming; red arrows indicate allicin-mediated activation of Nrf2/HO-1 signaling; green blunt-end lines indicate inhibition of ROS accumulation and NLRP3 assembly by Nrf2/HO-1; purple arrows indicate proximity-induced autocatalytic cleavage of pro-caspase-1 within the assembled inflammasome complex.

## 4. Cross-System Anti-Inflammatory Patterns of Allicin

### 4.1. Shared Regulatory Features of Allicin Across Organ Systems

**Figure 3:**
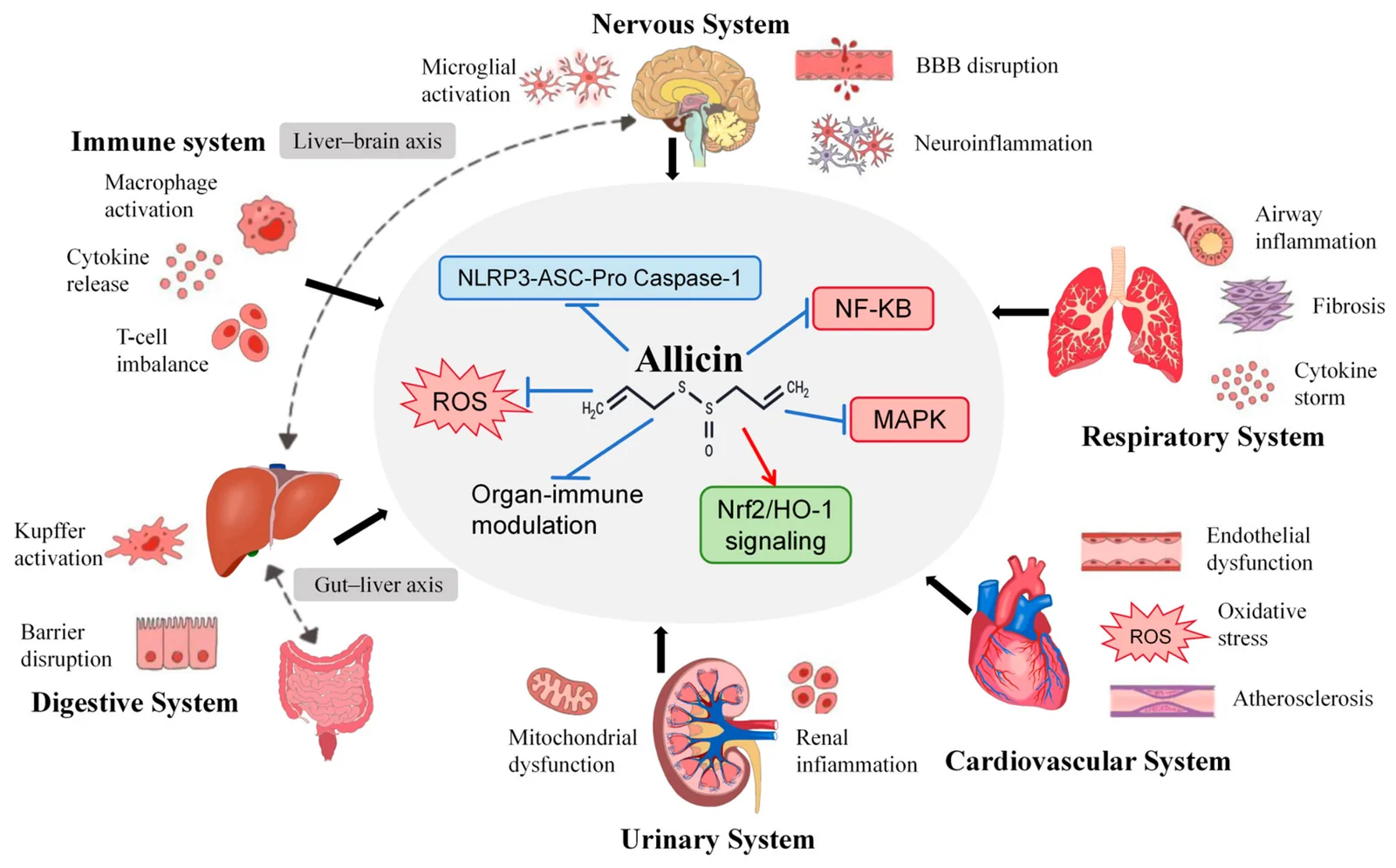
Overview of allicin’s multi-system anti-inflammatory activity.

Despite differences in the triggers and pathological processes of inflammatory diseases across organ systems, evidence from in vitro and animal models indicates that allicin exhibits fairly consistent anti-inflammatory effects across multiple disease models. Among these, suppression of NF-κB signaling, inhibition of the NLRP3 inflammasome, and activation of the Nrf2/HO-1 axis appear to be the most consistently reported patterns (Figure 3).

Suppression of the NF-κB pathway has been observed in models involving the digestive, respiratory, cardiovascular, urinary, and nervous systems. The major changes reported include inhibition of IκBα degradation and reduced p65 nuclear translocation, accompanied by decreased expression of pro-inflammatory cytokines such as TNF-α, IL-1β, and IL-6. This apparent cross-system consistency may be related to the broad cysteine-reactive properties of allicin. Proteomic studies have identified 332 S-thioallylated protein targets in human Jurkat T cells [107], including signaling kinases, transcription factors, and metabolic enzymes. This broad reactivity may partly explain the recurring pathway-level effects reported across different disease models [107].

The inhibitory effect of allicin on the NLRP3 inflammasome has been reported mainly in cardiovascular, neurological, digestive, and metabolic disease models. The NLRP3–ASC–pro-caspase-1 complex represents a well-established convergence point for anti-inflammatory intervention, including by natural compounds [109,110]. By reducing ROS accumulation and interfering with NLRP3 inflammasome assembly [26,96], allicin has been reported to inhibit caspase-1 activation and limit the maturation and release of IL-1β and IL-18 [55], which may reduce downstream inflammatory signal amplification. These effects have often occurred alongside activation of Nrf2 and reduced oxidative stress. In a myocardial injury model, siRNA-mediated Nrf2 silencing weakened the inhibitory effect of allicin on NLRP3 protein expression, suggesting a potential regulatory relationship between the Nrf2/HO-1 pathway and NLRP3 activation [55].

The Nrf2/HO-1 axis has been repeatedly implicated in allicin-mediated responses across multiple organ systems. Rather than functioning independently, its antioxidant effects may interact with inflammatory signaling by limiting oxidative stress, which can contribute to the persistence and amplification of inflammation [15,111].

Allicin has also been reported to affect immune cell function through several mechanisms, including modulation of the Treg/Th17 balance [112] and reduction in neutrophil infiltration as reflected by decreased MPO activity [29]. In addition, allicin downregulates intercellular adhesion molecule-1 (ICAM-1) expression, which may reduce inflammatory cell adhesion to vascular endothelial cells and limit their recruitment to sites of inflammation [113]. These immunomodulatory effects may contribute to the anti-inflammatory actions of allicin in chronic inflammatory conditions such as non-alcoholic steatohepatitis and atherosclerosis.

The apparent consistency of allicin’s effects on NF-κB, NLRP3, Nrf2/HO-1, and immune cell function across different organ systems should be interpreted with caution. Publication bias may contribute to this apparent consistency, while the predominance of studies focusing on individual signaling pathways and acute models, particularly LPS-stimulated macrophages and rodent models, limits conclusions regarding mechanistic specificity and clinical relevance. Given the broad protein reactivity associated with allicin-mediated S-thioallylation, the observed effects on individual pathways may also reflect broader cellular responses rather than selective pathway targeting. Future studies should examine multiple pathways within the same disease model, use well-characterized allicin preparations, and incorporate models that better reflect chronic inflammatory conditions. The available evidence supports the plausibility of a multi-pathway anti-inflammatory action of allicin, but its underlying mechanisms have not yet been fully established.

Blue blunt-end lines indicate direct inhibition by allicin of NLRP3 inflammasome, NF-κB, MAPK, ROS accumulation; red arrows indicate allicin-mediated activation of Nrf2/HO-1 signaling; gray dashed arrows indicate indirect inter-organ communication via the liver–brain axis and gut–liver axis. Through these mechanisms, allicin exerts multi-system anti-inflammatory protection across the nervous, respiratory, cardiovascular, urinary, and digestive systems.

### 4.2. System-Associated Targets and Regulatory Patterns Across Organ Systems

While allicin’s anti-inflammatory effects commonly involve suppressing NF-κB signaling, inhibiting the NLRP3 inflammasome, and activating the Nrf2/HO-1 axis, various organ systems appear to display distinct regulatory features. These differences may be related not only to variations in local inflammatory microenvironments but also to the dominant pathological processes involved in different diseases (Table 2). For example, the prominence of LXRα/ABCA1-mediated cholesterol efflux in cardiovascular models [47,114] is consistent with the central role of lipid metabolism in atherosclerosis, while the recurrence of mitochondrial quality-control mechanisms in cardiovascular and neurological tissues may reflect their high dependence on oxidative phosphorylation. However, none of these patterns has been systematically compared across organ systems within a single study, and the outcome measures have varied among the studies. Whether these patterns reflect genuine tissue specificity or differences in research focus therefore remains unresolved and warrants future cross-system comparative studies.

## 5. Challenges in Clinical Translation and Future Perspectives

### 5.1. Chemical Instability and Pharmacokinetic Limitations

Although allicin has shown anti-inflammatory activity in a variety of inflammatory disease models, its clinical translation has long been limited by a combination of factors, including pharmacokinetic drawbacks, chemical instability, difficulties in standardizing allicin preparations, variability among commercial garlic products, manufacturing challenges, and the limited number of human clinical trials conducted to date. As a highly reactive thiosulfinate compound, allicin is sensitive to temperature, pH, and solvent conditions. Allicin stability in aqueous solution is influenced by multiple factors, including pH, solvent composition, temperature, oxygen exposure, and concentration. Although allicin may be relatively more stable under mildly acidic conditions (pH 5–6), its stability is highly context-dependent and can vary substantially with the physicochemical conditions of the solution [115]. At physiological temperature (37 °C), allicin has been reported to have a half-life of less than 1 min [116], and its inherent instability remains one of the factors limiting its clinical application. During processing, storage, and in vivo exposure, allicin readily undergoes chemical decomposition into secondary organosulfur degradation products, such as diallyl disulfide (DADS) and diallyl trisulfide (DATS) [116], resulting in limited systemic exposure to the parent compound [13].

After entering the body, allicin is rapidly metabolized through reactions with GSH and other thiol-containing molecules, generating the enzymatically derived metabolite allyl methyl sulfide (AMS), in addition to non-enzymatic decomposition products such as DADS [117,118,119]. Human studies have shown that intact allicin is rarely detectable in biological samples such as blood and urine following oral garlic consumption. In most cases, systemic exposure can only be estimated indirectly through volatile metabolites such as AMS [117]. Although some of these metabolites retain biological activity, their anti-inflammatory effects and mechanisms of action may differ substantially from those of allicin, and their relative potency compared with allicin has not been consistently established across experimental models. Therefore, these compounds should not be considered direct substitutes for allicin when interpreting or comparing biological activity [120].

In addition to rapid degradation and metabolism, poor oral bioavailability further limits the therapeutic application of allicin. Allicin is not naturally present in intact garlic cloves but is generated from its precursor alliin through alliinase-catalyzed conversion. Because alliinase activity is reduced under gastric acidic conditions (pH < 3.5), allicin formation in the stomach is greatly restricted [121]. Furthermore, evaluations of commercially available enteric-coated garlic preparations have shown that, under simulated gastrointestinal conditions, most of the products tested released substantially less allicin than expected from their labeled content [121]. Chemical instability [14,115], rapid metabolism [118,121], and low oral bioavailability [121] may thus limit the ability of allicin to maintain effective concentrations in target tissues, and collectively represent important barriers to its clinical development and pharmaceutical translation. However, emerging formulation and delivery strategies, including nanoformulations, encapsulation systems, and other approaches designed to improve allicin stability, bioavailability, and targeted delivery, are being actively investigated to overcome these limitations.

These physicochemical and pharmacokinetic properties also complicate the interpretation of the preclinical evidence discussed above. Throughout Section 2, Section 3 and Section 4, the term “allicin” refers to the compound or preparation administered in the original studies. However, given its rapid degradation and reactivity, the observed effects may result from allicin itself, its degradation products, or their combined actions. In addition, the purity, stability, and actual concentration of allicin at the time of administration were not consistently characterized across studies. These factors should therefore be considered when interpreting the mechanistic findings described in the preceding sections.

### 5.2. Limitations of the Current Evidence

The apparent consistency of allicin’s effects on NF-κB, NLRP3, Nrf2/HO-1, and immune cell function across different disease models should be interpreted with caution. Publication bias may contribute to this apparent coherence [122], while the predominance of pathway-focused studies, often based on LPS-stimulated cells or rodent models, limits mechanistic specificity and the extrapolation of these findings to chronic inflammatory diseases in humans [123]. In addition, the broad thiol reactivity of allicin raises the possibility that some observed changes in signaling pathways reflect broader cellular effects rather than specific pathway targeting [107]. The available evidence thus supports the mechanistic plausibility of allicin’s multi-pathway anti-inflammatory activity, but does not yet establish a unified or definitive mechanism.

A further limitation of the current preclinical literature is the lack of consistent methodological rigor. Many studies rely on a single dose or treatment schedule, making it difficult to assess dose–response relationships and temporal effects. Mechanistic conclusions are also frequently based on changes in pathway-related markers without sufficient causal validation, such as genetic loss-of-function or rescue experiments [124]. In addition, sample sizes are often limited, and standardized procedures for allicin preparation, together with its purity, stability, and actual concentration at the time of administration, are not consistently reported across studies. These factors reduce the strength and reproducibility of the mechanistic conclusions and should be addressed in future studies.

### 5.3. Current Clinical Evidence

A critical gap exists between the extensive preclinical evidence reviewed above and the clinical literature: to date, no randomized controlled trials have evaluated purified, isolated allicin in humans. This gap is a direct consequence of allicin’s chemical instability, which precludes formulation of a stable, standardized oral allicin product. Instead, essentially all existing human data derive from whole garlic, garlic powder, or garlic extract supplements, in which allicin is one of several organosulfur components and is generated in situ from alliin via alliinase activity only after ingestion. Critically, the resulting allicin exposure is highly inconsistent across products: bioequivalence assays indicate that allicin release from different commercial garlic preparations ranges from approximately 22% to over 100% relative to a raw-garlic reference standard, depending on formulation (enteric-coated versus non-enteric tablets, powder capsules) and co-ingested meal composition, and is rarely, if ever, quantified in clinical trials [117]. Importantly, in the 16 randomized controlled trials included in the meta-analysis, inflammatory biomarkers were not consistently defined as primary outcomes. In several studies, markers such as CRP, TNF-α, and IL-6 were assessed as secondary or exploratory outcomes, as the original trials primarily focused on other endpoints, including blood pressure, endothelial function, lipid profiles, or vascular outcomes [125]. Therefore, the findings regarding inflammatory biomarkers should be interpreted with some caution, as some studies may not have been specifically powered to detect changes in these markers. Consequently, the extensive mechanistic and multi-system evidence for allicin discussed in Section 2, Section 3 and Section 4 currently rests entirely on preclinical models, and the causal relevance of these pathways to human inflammatory disease remains to be established through appropriately designed, allicin-quantified clinical trials.

### 5.4. Future Perspectives

#### 5.4.1. Multi-Omics Approaches and Inter-Organ Communication

Future research should integrate transcriptomics, proteomics, metabolomics, and epigenomics to characterize changes in inflammatory signaling following allicin treatment. In particular, chemical proteomics and site-specific mapping of S-thioallylation should be prioritized to identify direct protein targets of allicin and distinguish primary molecular interactions from downstream signaling changes. Such approaches could help clarify the molecular basis and pathway specificity of allicin’s anti-inflammatory effects. Single-cell RNA sequencing and spatial transcriptomics may further help clarify how allicin regulates different inflammatory cell populations and reveal its actions in complex inflammatory microenvironments [126].

The effects of allicin on the gut microbiota suggest that inter-organ communication networks, including the gut–liver, gut–brain, and gut–lung axes, may contribute to its anti-inflammatory activity in different disease settings. Future studies should further investigate how allicin influences inflammatory responses across different organs through the microbiota–metabolite–immune axis. Such studies may help improve our understanding of intervention strategies for inflammatory comorbidities involving multiple organ systems.

#### 5.4.2. Novel Drug Delivery Systems

Chemical instability, rapid metabolism, and low bioavailability are major factors limiting the clinical application of allicin. To address these issues, the development of novel delivery systems with improved stability, bioavailability, and tissue-targeting capacity is of considerable interest. Improving the delivery of allicin may represent an important step toward its clinical translation [120,127].

In recent years, liposomes, poly(lactic-co-glycolic acid) nanoparticles, lipid nanoparticles, and other nanocarrier platforms have been explored to improve the pharmacokinetic properties of allicin [120,127,128]. Because of its lipophilic nature, allicin may be compatible with lipid-based nanocarriers, and lipid encapsulation has been reported to reduce degradation and improve release stability under in vitro conditions [120]. Preclinical nanoparticle-based delivery systems have also shown potential to improve allicin stability and controlled release, although evidence that these approaches increase systemic exposure, circulation time, or target-tissue bioavailability in vivo remains limited [120,127]. Thus, nanodelivery technologies and controlled-release formulations represent promising strategies for addressing the instability and short half-life of allicin, but their ability to overcome these pharmacokinetic limitations and improve tissue exposure in vivo requires further pharmacokinetic and translational validation.

For neurological diseases, the BBB remains a major obstacle to drug delivery. Allicin possesses physicochemical properties that may be compatible with BBB penetration; however, direct experimental evidence demonstrating effective transport of intact allicin across the BBB remains limited [129]. Future studies may combine allicin with brain-targeted nanocarriers, receptor-mediated transport strategies, or stimulus-responsive delivery platforms to improve BBB penetration and increase drug accumulation in brain tissue [128]. Such approaches may provide additional opportunities for the treatment of neuroinflammatory disorders, including ischemic stroke, and Alzheimer’s disease.

#### 5.4.3. High-Quality Clinical Trials

Despite numerous preclinical studies highlighting allicin’s anti-inflammatory potential, high-quality clinical evidence remains scarce. As noted above, the limited clinical data currently available have been generated almost exclusively from garlic-derived preparations rather than purified allicin, and allicin exposure has rarely been directly quantified [125]. Consequently, future clinical research should prioritize standardized, purified, or well-characterized allicin formulations that allow accurate quantification of allicin exposure [117], rather than relying on garlic preparations with undefined or variable allicin content. Before efficacy trials can be meaningfully designed, further studies are needed to establish standardized quality-control measures for allicin formulations and to characterize the pharmacokinetics and pharmacodynamics of purified allicin in humans, define dose–response relationships and the optimal route of administration, and evaluate long-term safety and potential drug–drug interactions [130,131,132]. Once these foundational data become available, multicenter randomized controlled trials could be designed to evaluate the efficacy and safety of allicin in chronic inflammatory diseases, such as inflammatory bowel disease, chronic obstructive pulmonary disease, atherosclerosis, and diabetic nephropathy. Such trials should systematically compare different formulations, dosing regimens, and, where justified by prior evidence, combination treatment strategies to support the development of standardized clinical protocols.

Investigating the potential of allicin in combination with existing anti-inflammatory therapies may also be of interest, but such studies should be considered only after its pharmacokinetic, pharmacodynamic, safety, and drug-interaction profiles have been adequately characterized [130,131]. Addressing these fundamental gaps is a prerequisite for rigorous clinical evaluation and translation, rather than a guarantee of therapeutic success.

## 6. Conclusions

Inflammation plays a central role in the development and progression of many diseases. Natural products have therefore attracted increasing interest as potential anti-inflammatory agents because they can act on multiple targets, and some, including allicin, have been associated with a relatively favorable safety profile in the specific contexts studied to date, although this cannot be generalized across the broader and highly heterogeneous category of natural products. Allicin, the major bioactive sulfur-containing compound derived from garlic, has demonstrated anti-inflammatory activity in a variety of preclinical models involving the digestive, respiratory, cardiovascular, urinary, and nervous systems. These effects have been linked primarily to the inhibition of NF-κB signaling and NLRP3 inflammasome activation, together with activation of the Nrf2/HO-1 antioxidant pathway.

However, the mechanisms underlying the effects of allicin are not identical across different inflammatory conditions. Beyond these commonly reported pathways, allicin appears to engage additional regulatory mechanisms that depend on the disease context and tissue environment. For example, regulation of the gut microbiota–gut–liver axis has mainly been described in digestive diseases, whereas the STAT3/PIM1 axis and the miR-455-3p/claudin-4 pathway have been reported in respiratory disorders. In cardiovascular diseases, studies have highlighted LXRα/ABCA1-mediated cholesterol efflux and mitochondrial protection, while research in neurological disorders has implicated the PKC-δ/PLS3 mitophagy axis and OTUD1-mediated regulation of Nrf2. Whether these differences represent genuine tissue-specific effects of allicin or simply reflect differences in the current research focus remains unclear.

Despite the growing body of preclinical evidence, several obstacles continue to limit the clinical development of allicin. Its chemical instability, rapid metabolism, and low oral bioavailability remain important challenges. More importantly, high-quality clinical studies evaluating purified allicin are scarce, and the available evidence from garlic-derived preparations cannot necessarily be directly attributed to allicin itself. Further studies using chemical proteomics, multi-omics approaches, and improved delivery strategies may help identify its direct molecular targets and clarify its pharmacological properties. Well-designed randomized controlled trials of purified allicin will also be essential to establish its safety and determine whether the anti-inflammatory effects observed in experimental models can be translated into meaningful clinical benefits.

Current evidence supports allicin as a promising anti-inflammatory candidate at the preclinical level. However, its therapeutic value in humans has not yet been established, and further mechanistic, pharmacokinetic, and clinical studies are required before its potential can be translated into clinical practice.

## Figures and Tables

**Table 1:** Allicin and inflammatory diseases.

System	Disease	Model	Effective Dose	Route of Administration	Mechanisms	Pathway	Reference
Digestive system	Inflammatory bowel disease (IBD)	IL-1β-stimulated Caco-2 cells and IBD animal model	Allicin 30 mg/kg, Mesalazine 30 mg/kg, Sulfasalazine 100 mg/kg	oral gavage	↓IL-8, ↓NF-κB p65, ↓TNF-α, ↓IL-1β, ↑IL-4	p38/JNK; NF-κB	[17]
		LPS-treated IPEC-J2 cells	Allicin 2 μg/mL	cell-based administration	↑p-Nrf2, ↑HO-1, ↑ZO-1, ↑occludin, ↑claudin-1	Nrf2/HO-1	[18]
		Experimental intestinal oxidative injury model	SeMet 0.4 mg/kg Allicin 20 mg/kg ML385 30 mg/kg	oral gavage; intraperitoneal injection	↑Nrf2, ↓inflammation, ↓oxidative stress	Nrf2	[19]
		TNF-α-stimulated HT-29 and Caco-2 cells	Allicin 20 μM	cell-based administration	↓IL-1β, ↓IL-8, ↓IP-10	IκBα/NF-κB	[20]
	Acrylamide-induced intestinal injury	Acrylamide-induced intestinal injury model	Allicin optimal dose 50 mg/kg	oral gavage	↑SCFAs, ↓inflammation, improved barrier function	TLR4/MyD88/NF-κB; gut microbiota–SCFA axis	[21]
	Alcoholic fatty liver disease (AFLD)	Ethanol-induced liver injury model	Allicin optimal dose 20 mg/kg	oral gavage	↑GSH, ↑CAT, ↓CYP2E1, ↓SREBP-1	CYP2E1; antioxidant signaling	[22]
		Alcohol-fed mouse model	Allicin 5 mg/kg	oral gavage	Gut microbiota remodeling; ↓TNF-α, ↓IL-1β, ↓IL-6	LPS-CD14-TLR4/NF-κB	[24]
	Metabolic dysfunction-associated steatotic liver disease (MASLD)	High-fat, high-fructose diet model	Allicin 20 mg/kg	oral gavage	↓TNF-α, ↓IL-1β, ↓IL-6; improved gut microbiota composition; ↑SCFAs	Rev-erbα/Bmal1; gut microbiota–gut–liver axis	[25]
	Acrylamide-induced hepatotoxicity	Acrylamide-treated BRL-3A cells and Kupffer cells	Allicin 20 mg/kg	oral gavage	↓ROS, ↓ER stress, ↓IL-1β, ↓IL-18	CYP2E1; IRE1α/TRAF2/p-ASK1/XBP-1s; MAPK/NF-κB/NLRP3	[26]
	Hepatic ischemia/reperfusion injury	Hepatic I/R model	Allicin optimal dose 30 mg/kg	oral gavage	↓phospho-p38, ↓phospho-p65, ↓TNF-α, ↓IL-1β	PPARγ/IRAK-M/TLR4	[27]
	Cyclophosphamide-induced hepatotoxicity	Cyclophosphamide-induced liver injury model	Allicin 10 mg/kg	oral gavage	↓iNOS/NOS2, ↓pro-inflammatory cytokines	Nrf2/ARE	[28]
Respiratory system	Acute lung injury (ALI) / Acute respiratory distress syndrome (ARDS)	LPS-induced septic lung injury model	Allicin optimal dose 100 μg/mL, injection volume: 20 mL/kg	intraperitoneal injection	↓TNF-α, ↓IL-6, ↓IL-1β, ↓Caspase-3, ↓Caspase-9	TLR4/MyD88/NF-κB	[29]
		LPS-induced ALI model	Allicin 10 mg/kg	tail vein injection	↓TNF-α, ↓IL-6, ↓IL-1β, ↓MPO, ↑Claudin-4	miR-455-3p/Claudin-4	[30]
	Pulmonary fibrosis (PF)	Experimental pulmonary fibrosis model	Allicin 50 mg/kg	oral gavage	↓inflammatory infiltration, ↓ECM deposition, ↓collagen contraction	AMPK/TGF-β1/Smad3	[31]
	Invasive pulmonary aspergillosis (IPA)	Aspergillus fumigatus infection model	Allicin 5 mg/kg	intravenous injection	↓ROS, ↓inflammatory cytokines, ↓LC3B-II	ROS/NLRP3; LC3B-II-mediated autophagy	[35]
	Asthma	Experimental asthma model	Allicin 10 mg/kg or 20 mg/kg	intraperitoneal injection	↓IL-4, ↓IL-5, ↓IL-13, ↓Eosinophil infiltration	STAT3/PIM1	[36]
	Laryngotracheal stenosis (LTS)	Experimental laryngotracheal stenosis model / allicin-coated tracheal tube model	Allicin 10 μg	PDA-mediated coating technology for localized contact-triggered release	↓TNF-α, ↓IL-1β, ↓IL-6, ↓collagen deposition	Fibroblast activation; bacterial biofilm inhibition	[40]
Cardiovascular system	Atherosclerosis (AS)	Endothelial cell injury model	Allicin 40 μg/mL	cell-based administration	↓ROS, ↓inflammatory cytokines, ↑mitochondrial function	Mitochondrial protection	[45]
		High-fat diet-induced coronary atherosclerosis mouse model	Allicin 10 mg/kg	oral gavage	↑NO, ↑EPC survival, ↑EPC migration	eNOS/Nrf2/HO-1	[46]
		THP-1 macrophage-derived foam cells	Allicin 5 μg/L	cell-based administration	↑ABCA1, ↑cholesterol efflux	PPARγ/LXRα/ABCA1	[47]
	Myocardial ischemia/reperfusion injury (MI/R)	Myocardial I/R model	Allicin 1.88 mg/kg	oral gavage	↓p-GRK2, ↓p-CaMKII, ↓p-PLC-γ, ↓p-IP3R, ↓Ca^2+^ overload	PI3K/GRK2/PLC-γ/IP3R	[49]
	Doxorubicin-induced cardiotoxicity	DOX-induced cardiotoxicity model	Allicin optimal dose 20 mg/kg	oral gavage	↑GSH, ↓NO, ↓MDA, ↓IL-1β, ↓TNF-α	Antioxidant signaling; Caspase-3	[52]
	Sepsis-associated myocardial dysfunction (SAMD)	LPS-injured cardiomyocytes	Allicin optimal dose 50 µM	cell-based administration	↑HO-1, ↑SOD, ↓MDA, ↓IL-1β, ↓NLRP3	Nrf2/HO-1/NLRP3	[55]
	Pulmonary arterial hypertension (PAH)	Experimental PAH model	Allicin 16 mg/kg	oral gavage	↓TNF-α, ↓IL-6, ↓IL-1β, ↓α-SMA	TGF-β/α-SMA	[57]
	Diabetic cardiomyopathy (DC)	Diabetic cardiomyopathy rat model	Allicin 40 mg/kg + artemisinin (combination)	oral gavage	↓NF-κB p65, ↓p-NF-κB p65, ↓ROS, ↓inflammatory cytokines	NF-κB; NADPH oxidase	[59]
Urinary system	Sepsis-associated acute kidney injury (S-AKI)	CLP-induced sepsis mouse model	20 mg/kg	intraperitoneal injection	↓SCr, ↓BUN, ↓UALB, ↓KIM-1, ↓NGAL	Nrf2/HO-1	[62]
	Renal ischemia/reperfusion injury (Renal I/R)	Renal I/R injury model	Allicin 50 mg/kg	intraperitoneal injection	↑SOD, ↓MDA, ↑IL-4, ↑IL-10, ↓TNF-α	Antioxidant signaling	[64]
	Cisplatin-induced nephrotoxicity	CDDP-induced nephrotoxicity rat model	Allicin 10 mg/kg; ascorbic acid 20 mg/kg	oral gavage	↑GSH, ↑SOD, ↓MDA, ↓NO, ↓TNF-α		[65]
	Gentamicin-induced nephrotoxicity	Gentamicin-induced nephrotoxicity model	Allicin 50 mg/kg	oral gavage	↑GSH, ↑SOD, ↓MDA, ↓MPO, ↓TNF-α		[66]
	Metabolic syndrome-associated kidney injury	Metabolic syndrome model	Allicin 16 mg/kg	oral gavage	↓IL-1β, ↓IL-6, ↓TNF-α, ↑IκB, ↑Nrf2	Nrf2/HO-1; NF-κB	[15]
	Diabetic nephropathy (DN)	Diabetic nephropathy model	Allicin 16 mg/kg	oral gavage	↓CRP, ↓IL-1, ↓IL-6, ↓IL-18, ↓TNF-α	TGF-β1	[67]
	Urinary tract infection (UTI)	Escherichia coli-induced UTI model	Allicin optimal dose 50 mg/kg	intraperitoneal injection	↓IL-6, ↓IL-1β	MALT1/AKT/NF-κB	[69]
Nervous system	Traumatic brain injury (TBI)	Controlled cortical impact (CCI) model	Allicin optimal dose 30 mg/kg	intraperitoneal injection	↓TNF-α, ↓IL-1β, ↓IL-6, ↓ROS, ↓NLRP3	PKC-δ/PLS3; TLR4/NLRP3	[72]
	Spinal cord injury (SCI)	Experimental SCI model	Allicin optimal dose 50 mg/kg	intraperitoneal injection	↑Nrf2, ↓Pro-inflammatory cytokines	Nrf2	[73]
	Intracerebral hemorrhage (ICH)	Experimental ICH model	Allicin 50 mg/kg	intraperitoneal injection	↓IL-6, ↓CXCL2, ↓neutrophil infiltration, ↓microglia/macrophage accumulation	Neuroimmune regulation	[76]
	Subarachnoid hemorrhage (SAH)	Experimental SAH model	Allicin 70 mg/kg	intraperitoneal injection	↓MDA, ↑SOD, ↑GSH, ↓Caspase-3	Antioxidant signaling	[77]
	Ischemic stroke	Experimental ischemic stroke model	Allicin 50 mg/kg	intraperitoneal injection	↓TNF-α, ↓MPO, ↓infarct volume	Anti-inflammatory signaling	[79]
	Cerebral ischemia/reperfusion injury	Cerebral I/R injury model	Allicin 50 mg/kg	intraperitoneal injection	↑Nrf2, ↓mitochondrial oxidative stress	OTUD1/Nrf2	[80]
	Alzheimer’s disease (AD)	Experimental AD model	10 mg/kg	oral gavage	↑SOD, ↓MDA, ↓Bax, ↑Bcl-2	JNK/c-Jun	[84]
		Aluminum-copper-induced AD model	20 mg/kg	intraperitoneal injection	↓IL-1β, ↓IL-6, ↓TNF-α, ↑GSH	Antioxidant signaling	[85]
	Age-related cognitive decline	Aging model	180 mg/kg	oral feed administration	↑Nrf2, ↓oxidative stress	Nrf2	[86]
	Hepatic encephalopathy (HE)	Acute liver failure model	Allicin optimal dose 200 mg/kg	oral gavage	↓TNF-α, ↓IL-1β, ↓oxidative stress markers	Anti-inflammatory; antioxidant signaling	[88]
	Acrylamide-induced neurotoxicity	Acrylamide neurotoxicity model	Allicin 20 mg/kg	oral gavage	↑GSH, ↓GSSG, ↑ACh, ↑DA, ↓AChE	Antioxidant signaling	[90]
	Depressive-like behavior	Experimental depression model	Allicin optimal dose 50 mg/kg	intraperitoneal injection	↓ROS, ↓MDA, ↑SOD, ↓NLRP3	Nrf2; NLRP3	[91]

Notes: ↑ indicates increase/upregulation; ↓ indicates decrease/downregulation.

**Table 2:** Representative organ-associated anti-inflammatory features of allicin.

Organ System	Representative Organ-Specific Pathways	Associated Physiological/Cellular Processes
Digestive system	Rev-erbα/Bmal1; CYP2E1	Gut microbiota–gut–liver axis;
Respiratory system	miR-455-3p/claudin-4; STAT3/PIM1; AMPK/TGF-β1/Smad3	Alveolar epithelial barrier integrity
Cardiovascular system	LXRα/ABCA1; eNOS/Nrf2/HO-1	Mitochondrial membrane potential maintenance
Urinary system	MALT1/AKT/NF-κB	IL-4/IL-10-mediated shift in cytokine expression
Nervous system	PKC-δ/PLS3; OTUD1/Nrf2 axis	Cardiolipin-dependent mitophagy

## Data Availability

No new data were created or analyzed in this study. Data sharing is not applicable to this article.

## Abbreviations

The following abbreviations are used in this manuscript:

ABCA1	ATP-binding cassette transporter A1
ACh	acetylcholine
AChE	acetylcholinesterase
AD	Alzheimer’s disease
AFLD	alcoholic fatty liver disease
AKI	acute kidney injury
AKT	protein kinase B
ALI	acute lung injury
AMPK	AMP-activated protein kinase
AMS	allyl methyl sulfide
ARE	antioxidant response element
ARDS	acute respiratory distress syndrome
AS	atherosclerosis
ASC	apoptosis-associated speck-like protein containing a CARD
α-SMA	alpha-smooth muscle actin
BALF	bronchoalveolar lavage fluid
BBB	blood–brain barrier
BDNF	brain-derived neurotrophic factor
Bmal1	brain and muscle ARNT-like protein 1
BUN	blood urea nitrogen
CARD	caspase recruitment domain
CAT	catalase
CHOP	CCAAT/enhancer-binding protein homologous protein
CKD	chronic kidney disease
CLP	cecal ligation and puncture
CRP	C-reactive protein
CVD	cardiovascular disease
CXCL2	C-X-C motif chemokine ligand 2
CYP2E1	cytochrome P450 2E1
DA	dopamine
DADS	diallyl disulfide
DAMPs	damage-associated molecular patterns
DAT	dopamine transporter
DATS	diallyl trisulfide
DC	diabetic cardiomyopathy
DN	diabetic nephropathy
DOX	doxorubicin
Drp-1	dynamin-related protein 1
ECM	extracellular matrix
ELISA	enzyme-linked immunosorbent assay
eNOS	endothelial nitric oxide synthase
EPC	endothelial progenitor cell
ER	endoplasmic reticulum
ERK	extracellular signal-regulated kinase
Fis-1	fission protein 1
GCLC	glutamate-cysteine ligase catalytic subunit
GPx	glutathione peroxidase
GRP78	glucose-regulated protein 78
GSDMD	gasdermin D
GSH	glutathione
GSSG	glutathione disulfide
HE	hepatic encephalopathy
HO-1	heme oxygenase-1
HUVECs	human umbilical vein endothelial cells
IBD	inflammatory bowel disease
ICAM-1	intercellular adhesion molecule-1
ICH	intracerebral hemorrhage
IKK	IκB kinase
IκBα	inhibitor of nuclear factor-κB alpha
IL-1β	interleukin-1 beta
IL-4	interleukin-4
IL-5	interleukin-5
IL-6	interleukin-6
IL-8	interleukin-8
IL-10	interleukin-10
IL-13	interleukin-13
IL-18	interleukin-18
iNOS	inducible nitric oxide synthase
IP-10	interferon gamma-induced protein 10
IPA	invasive pulmonary aspergillosis
IRAK-M	interleukin-1 receptor-associated kinase M
IRE1α	inositol-requiring enzyme 1 alpha
IRI	ischemia/reperfusion injury
JNK	c-Jun N-terminal kinase
Keap1	Kelch-like ECH-associated protein 1
KIM-1	kidney injury molecule-1
LC3B-II	microtubule-associated protein 1 light chain 3 beta-II
LPS	lipopolysaccharide
LTS	laryngotracheal stenosis
LXRα	liver X receptor alpha
MALT1	mucosa-associated lymphoid tissue lymphoma translocation protein 1
MAPK	mitogen-activated protein kinase
MASLD	metabolic dysfunction-associated steatotic liver disease
MCP-1	monocyte chemoattractant protein-1
MDA	malondialdehyde
MEKK2	mitogen-activated protein kinase kinase kinase 2
Mfn-1	mitofusin-1
MI/R	myocardial ischemia/reperfusion
MK2	MAPK-activated protein kinase 2
MPO	myeloperoxidase
mRNA	messenger RNA
MS	metabolic syndrome
MyD88	myeloid differentiation primary response protein 88
NADPH	nicotinamide adenine dinucleotide phosphate
NF-κB	nuclear factor-kappa B
NGAL	neutrophil gelatinase-associated lipocalin
NLRP3	NOD-like receptor thermal protein domain associated protein 3
NO	nitric oxide
NOS2	nitric oxide synthase 2
NOx	nitric oxide metabolites
NQO1	NAD(P)H quinone oxidoreductase 1
Nrf2	nuclear factor erythroid 2-related factor 2
NSAIDs	non-steroidal anti-inflammatory drugs
Opa-1	optic atrophy protein 1
OTUD1	OTU deubiquitinase 1
p-ASK1	phosphorylated apoptosis signal-regulating kinase 1
p-CREB	phosphorylated cAMP response element-binding protein
p-Nrf2	phosphorylated nuclear factor erythroid 2-related factor 2
p38 MAPK	p38 mitogen-activated protein kinase
PAH	pulmonary arterial hypertension
PAMPs	pathogen-associated molecular patterns
PERK	protein kinase R-like endoplasmic reticulum kinase
PI3K	phosphoinositide 3-kinase
PIM1	proviral integration site for Moloney murine leukemia virus 1
PKA	protein kinase A
PKC-δ	protein kinase C delta
PLS3	plastin 3
PPARγ	peroxisome proliferator-activated receptor gamma
PRRs	pattern recognition receptors
Rev-erbα	nuclear receptor subfamily 1, group D, member 1
ROS	reactive oxygen species
SAH	subarachnoid hemorrhage
SAMD	sepsis-associated myocardial dysfunction
SCFAs	short-chain fatty acids
SCr	serum creatinine
SCI	spinal cord injury
siRNA	small interfering RNA
Smad3	SMAD family member 3
SOD	superoxide dismutase
SREBP-1	sterol regulatory element-binding protein 1
STAT3	signal transducer and activator of transcription 3
TBARS	thiobarbituric acid reactive substances
TBI	traumatic brain injury
TGF-β	transforming growth factor-beta
TGF-β1	transforming growth factor-beta 1
Th2	T helper 2 cell
TLR4	Toll-like receptor 4
TNF-α	tumor necrosis factor-alpha
TRAF2	tumor necrosis factor receptor-associated factor 2
Treg	regulatory T cell
TUNEL	terminal deoxynucleotidyl transferase dUTP nick end labeling
UALB	urinary albumin
UTI	urinary tract infection
XBP-1s	spliced X-box binding protein 1
4-HNE	4-hydroxynonenal
